# Evaluating Diet Quality of Canadian Adults Using Health Canada’s Surveillance Tool Tier System: Findings from the 2015 Canadian Community Health Survey-Nutrition

**DOI:** 10.3390/nu12041113

**Published:** 2020-04-16

**Authors:** Salma Hack, Mahsa Jessri, Mary R. L’Abbé

**Affiliations:** 1 Department of Nutritional Sciences, Faculty of Medicine, University of Toronto, Toronto, ON M5S 1A8, Canada; salma.hack@mail.utoronto.ca; 2 Food Nutrition and Health Program, The University of British Columbia, Vancouver, ON V6T 1Z4, Canada; mahsa.jessri@ubc.ca

**Keywords:** Canadian Community Health Survey-Nutrition 2015, Health Canada’s Surveillance Tool, Tier System, dietary intakes, nutrient profiling, diet quality, nutrition policy

## Abstract

The 2014 Health Canada’s Surveillance Tool, Tier System (HCST) is a nutrient profiling model developed to evaluate adherence of food choices to dietary recommendations. With the recent release of the nationally representative Canadian Community Health Survey-Nutrition (CCHS-N) 2015, this study used HCST to evaluate nutritional quality of the dietary intakes of Canadians in the CCHS-N. Dietary intakes were ascertained using 24-hour dietary recalls from Canadians adults ≥19 years (*N* = 13,605). Foods were categorized into four Tiers based on degree of adherence to dietary recommendations according to thresholds for sodium, total fat, saturated fats, and sugars. Tier 1 and Tier 2 represented “recommended foods”, Tier 3 represents foods to “choose less often”, and Tier 4 represented foods “not recommended”. Across all dietary reference intakes (DRI) groups, most foods were categorized as Tier 1 for Vegetable and Fruits (2.2–3.8 servings/day), Tier 2 for Grain Products (2.9–3.4 servings/day), Tier 3 for Milk and Alternatives (0.7–1 serving/day) or for Meat and Alternatives (1.1–1.6 servings/day). Consumption of foods from Tier 4 and “other foods” such as high fat/sugary foods, sugar-sweetened beverages, and alcohol, represented 24–26% and 21–23% kcal/day, for males and females, respectively. Canadians are eating more foods categorized as Tier 1–3, rather than Tier 4. Adults with the highest intakes of Tier 4 and “other foods” had lower intakes of macronutrients and increased body mass index. These findings can be used by policy makers to assist in identifying targets for food reformulation at the nutrient level and quantitative guidance to support healthy food choices.

## 1. Introduction

According to the most recent 2019 Global Burden of Disease report (GBD), dietary risk factors contributed to approximately 11 million deaths globally [1,2]. The GBD report indicated that among all modifiable risk factors, which include unhealthy diets, physical inactivity, tobacco, and alcohol consumption, dietary risk factors and tobacco use are the top two behavioral risk factors in Canada causing both death and disability; therefore action is needed to improve dietary habits [1,2,3,4].

To assess the nutritional quality of Canadian dietary intakes, Health Canada developed the 2014 Health Canada Surveillance Tool (HCST) Tier System, as a nutrient profiling tool used to determine the healthfulness of foods and beverages based on thresholds for nutrients of public health concern i.e., sodium, saturated fats, total fats, and sugars [5,6,7,8]. HCST classifies foods into four categories: Tier 1, Tier 2, Tier 3, and Tier 4 and an additional group of “other foods”. Tiers 1 and 2 represent foods that Canadians should choose to consume most often, these foods are in line with Eating well With Canada’s Food Guide (EWCFG) 2007; Tier 3 foods should be chosen less often, these foods are partially in line with EWCFG; while Tier 4 foods are items to limit, not in line with EWCFG (foods high in sodium, total fats, saturated fats and sugars) [5,6,7]. Additionally, Health Canada created the “other foods” category for foods with no serving recommendations, such as sugar sweetened beverages, confectionaries, or alcohol, based on dietary guidelines and thus these foods along with Tier 4 foods are foods to limit.

To our knowledge, no previous study has evaluated adherence of current dietary intakes of Canadians at the national level using the 2015 national nutrition survey data with the HCST recommendations, although an earlier study evaluated dietary intakes from the 2004 nutrition survey [6]. Considering the significant changes in dietary trends and food policies in the intervening 11 years (2004–2015), this study examines the nutritional quality of Canadian diets using the HCST nutrient profiling system and the latest national nutrition survey of Canadians.

The newly released 2015 CCHS-Nutrition provides the unique opportunity to update these evaluations, to understand current diet quality, and to assess trends in Canadians’ intakes. With the release of Canada’s 2019 Dietary Guidelines, Health Canada continues to recommend consuming and choosing foods low in sodium, sugars, and saturated fats [5,9]. New recommendations outlined in Canada’s 2019 Dietary Guidelines in conjunction with the HCST Tier system can provide evidence on dietary intakes of these nutrients in relation to foods and beverages consumed by Canadians. Dietary data reflecting the current nutritional quality of foods consumed by Canadians is lacking, hindering policy and decision makers from developing relevant nutrition guidelines and recommendations. This study aimed to investigate the diet quality of Canadian adults ≥19 years using the most recent 2015 CCHS-Nutrition, through application of 2014 HCST which assesses the nutritional quality of foods choices based on thresholds for nutrients to limit.

## 2. Materials and Methods

### 2.1. Experimental Design

The 2015 CCHS-Nutrition is a voluntary, nationally representative nutrition survey conducted by Statistics Canada. The 2015 CCHS-Nutrition survey is a cross-sectional design with three sampling stages. Dietary intake interviews were computer assisted using a five-step automated multi-pass method (AMPM), adapted and modified for the Canadian population from the United States Department of Agriculture (USDA). The survey was conducted January 2, 2015 to December 31, 2015, on all days of the week [10,11,12]. Two separate questionnaires were administered: a) 24-hour dietary recall, and b) a general health questionnaire to support the 24-hour dietary recall. This survey provides the most current nutrition data available on Canadians. The survey is nationally representative of twelve age–sex groups, which correspond to the Dietary Reference Intakes (DRI) groupings [10,11,12]. The final sample included 20,487 respondents, with a 61.6% response rate [10]. Detailed information on the sampling of respondents can be found in The Canadian Community Health Survey, Nutrition CCHS user-guide [10,11]. Socio-demographic and lifestyle characteristics were also collected using Statistics Canada’s survey questionnaires [10,11].

### 2.2. Subjects

Surveyed participants for 2015 CCHS-Nutrition were those residing in Canada’s 10 provinces for ≥1 year, excluding individuals living in: northern territories, on reserves, Aboriginal settlements, full-time members of the Canadian Armed Force, and institutionalized individuals [10]. For the purpose of these analyses we excluded anyone with no food reported and missing energy intake as defined by Statistics Canada [11], breastfeeding and pregnant women (*n* = 683). Single 24-hour dietary recalls of males and females ≤18 years (*n* = 6199) were removed from analyses to focus on dietary intakes of adults, leaving a total sample size of 13,605.

### 2.3. Classifying Foods in the 24-Hour Dietary Recall According to HCST 

The current version, the 2015 Canadian Nutrient File (CNF), was used to estimate the nutrient content of food [11,12]. The CNF contains the nutritional composition of 5690 unique Canadian foods commonly consumed, accounting for Canadian food fortification and composition regulations [5,10,12]. Health Canada and Public Health Agency of Canada developed the CNF/Canada’s Food Guide (CFG) classification, enabling linkage of CNF food codes to four Eating Well with Canada’s Food Guide (EWCFG) food groups and 21 subgroups [10]. Foods reported in the detailed single day 24-hour dietary recall were linked with CNF 2015 to determine their nutritional composition. HCST uses the nutritional quality of food items according to the nutrient composition in the CNF, which represents a standard reference amount for foods commonly consumed by Canadians [5,6]. Foods were then categorized based on the HCST Tier nutrient profiling system.

Health Canada developed upper and lower thresholds for the Tier system based on four nutrients of public health concern: sodium, saturated fats, total fats, and sugars [5,8,10]. Cut-points were derived from thresholds used for nutrient content claims, dietary reference intakes (DRI) for macronutrients, and nutrition standards for foods in schools [5].

Lower thresholds for fats and sodium content were based on thresholds for foods in order to meet “low in” nutrient content claims for an amount of food commonly consumed in one sitting, termed the Reference Amount (RA) [5,6,7]. Foods categorized as Tier 1 must not exceed the lower thresholds: ≤3 g/RA for fat, ≤140 mg/RA for sodium, and ≤6 g/RA for sugar [5]. As there is no daily value (DV) for sugar, upper and lower thresholds for sugar were determined from the recommendation by the Institute of Medicine (IOM) [5]. The 15% DV of sodium (>360 mg/RA), total fats (>10 g/RA), saturated fats (>2 g/RA), and sugars (>19 g) were set as the upper thresholds [5]. Foods from the 24-hour dietary recalls were categorized as Tier 1 when nutrient values did not exceed any of the three lower thresholds for fat, sugar, and sodium; Tier 2 foods could exceed one or two lower thresholds but no upper thresholds. Tier 3 foods were foods with nutrient contents above all lower thresholds (i.e., sodium, sugar, and total fat), and may exceed one upper threshold. Tier 4 represents foods that exceed ≥2 upper thresholds, however special consideration was given to foods belonging to the Meat and Alternative, and Milk and Alternative categories as they naturally have higher saturated fat content. For example, Tier 2 and Tier 3 legumes, nuts and seed, and others contain increased amounts of natural oils, therefore these foods result in a shift in Tiers i.e., from Tier 3 to Tier 2 if the lower threshold for sodium and sugar and upper thresholds for saturated fat are not exceeded. [5]. Additional adjustments for foods based on directional statements from CFG can be found in HCST [5].

### 2.4. “Other Foods” and Beverages

According to the CNF/CFG classification, nine groups of foods could not be classified according to Tiers 1–4 [5]. Of these groups, five categories of foods were grouped as “other foods” representing those not recommended by EWCFG. These groups are: (1) saturated and/or trans-fats and oils; (2) high-fat and high sugar foods such as, candies, chocolates syrups; (3) high calorie beverages ≥40 kcal/100g; (4) low calorie beverages <40 kcal/100g; and (5) alcoholic beverages [5,6,7].

Foods classified as Tier 4 and “other foods” and beverages have no acceptable Canada Food Guide Serving [5,6,7,13]. These foods have directional statements as food items to limit (similar to HCST Tier 4 foods such as: cakes, muffins, chocolates potato chips, or fruit flavored drinks etc.). This study determined energy contributions of Tier 4 and “other foods” and beverages outlined above for adults by DRI age–sex groups as done previously [6].

### 2.5. Statistics

Analyses were completed using Statistical Analysis Software (SAS) version 9.4 (SAS Institute Inc., Cary, NC, USA). Bootstrap balanced repeated replication with 500 repeats was used to estimate population parameters i.e., confidence intervals, standard errors, and coefficients of variation. Survey weights provided with the master files were used for *N* = 13,605 adults, to ensure samples from 2015 CCHS-Nutrition remain nationally representative [10,11]. Dietary intakes were assessed according to DRI age–sex groupings and additional lifestyle measures, which included smoking, physical activity, and Body Mass Index (BMI). PROC SURVEYREG and PROC SURVEYLOGISTIC were used for continuous (e.g., servings from fruit and vegetables) and for categorical (e.g., lifestyle measures) analyses, respectively, adjusting for energy intake, age, and sex where appropriate. Results with two- tailed *p*-value ≤0.05 were reported as statistically significant.

### 2.6. Identification of Implausible Reporters

Previous studies have found participants with higher BMI (i.e., overweight and obese) tend to under-report socially undesirable foods e.g., high fat and sugar foods, sugar-sweetened beverages etc. This is recognized as selective misreporting [14]. In accordance with publications from the two cycles of CCHS (Jessri et al. (2015) and Garriguet et al. (2016)), we have categorized participants based on comparison of their estimated energy requirement (EER) to total energy expenditure (EER:TEE) [6,7,14,15,16,17,18]. EER was determined based on the equation developed by the Institute of Medicine (IOM) accounting for age, sex, BMI, and physical activity [19]. For individuals with no measured height and weight (due to refusal, surrounding conditions, or did not permit measurements) to determine BMI, USDA energy levels were assigned to adult participants to determine EER based on sex and physical activity levels for sedentary, low active, active, and very active [15,17,18,19]. Adults were categorized as under-reporters if energy intake (EI) was <70%, plausible if EI was 70–142%, and over-reporters if EI was >142% [6,7,15,16].

### 2.7. Approvals

The data in this study was completed as secondary analyses. Data analysis was performed at the Research Data Centre (RDC) of Statistics Canada, Toronto Ontario. A trained staff member at the Toronto RDC vetted data before release.

## 3. Results

This study included 13,605 Canadian adults (ages ≥19 years). Table 1 shows average intakes of EWCFG food groups (servings/day) categorized into Tiers 1–4. Across all DRI age–sex groups, daily intake of foods from Tier 1–3 were below recommendations for all food categories except for women 31–50 years who met the recommendations for Meat and Alternatives. The majority of EWCFG servings were reported from Grain Products (Tier 2: 2.9 to 3.4 servings/day), followed by Vegetables and Fruit (Tier 1: 2.2 to 3.8 servings/day), Meat and Alternatives (Tier 3: 1.1 to 1.6 servings/day), and Milk and Alternatives (Tier 3: 0.7 to 1 serving/day) for all DRI age–sex categories. Intakes of Meat and Alternatives among males decreased with age, with the highest intake reported as servings/day (Standard Error of the Mean (SEM); 2.69 (0.2) to 2.12 (0.1)) for males (19–30 years, to >70 years), and were mainly categorized as Tier 3. Consumption of Milk and Alternatives such as, dairy, cheeses, yogurt etc., was the lowest among males, however dietary intakes improved across age groups 31–50 years to >70 years (1.19 (0.1) to 1.45 (0.1)). Canadian’s average intakes of Vegetables and Fruit was below recommendations. Across DRI age–sex categories, from 19–30 years to >70 years the average number of servings/day of Vegetables and Fruit increased, with the exception of a slight decline among women 51–70 years to >70 years (4.91(0.2) to 4.7 (0.2) servings/day; Table 1).

**Figure 1:**
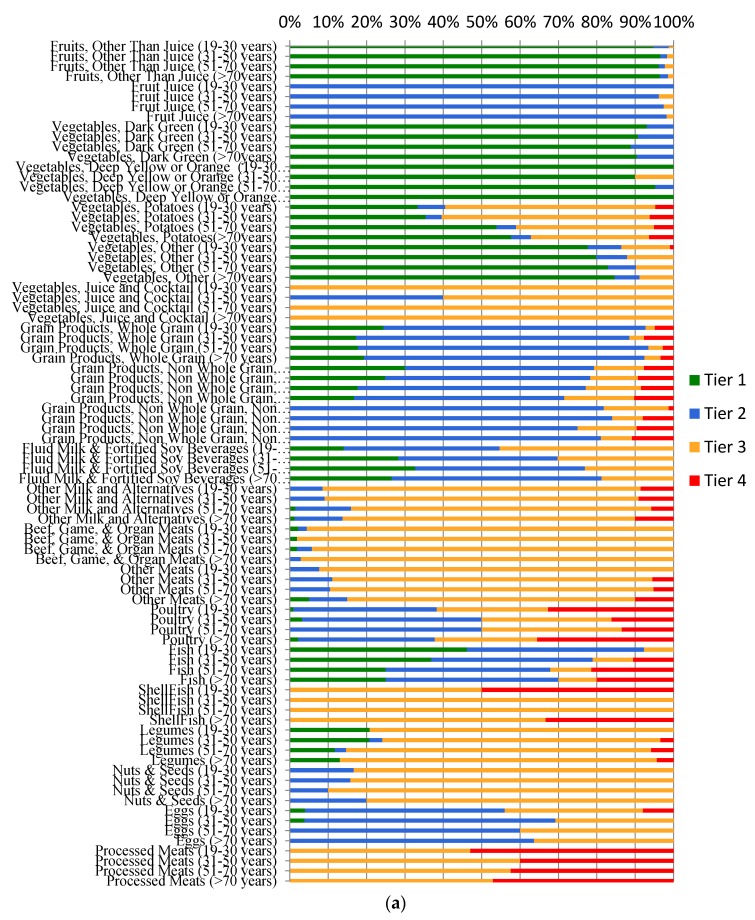

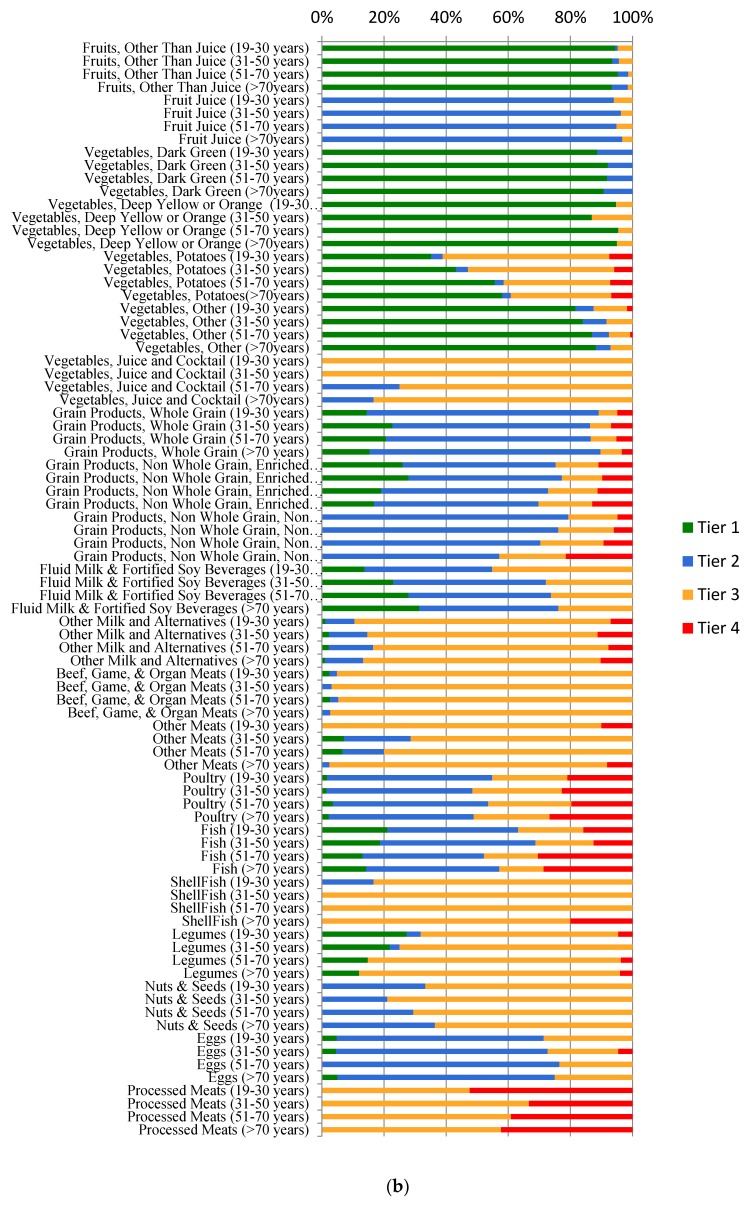
Weighted age-stratified analysis of foods consumed by food category based on the 2014 Health Canada’s Surveillance Tool, Tier system, among adults ≥19 years, *N* = 13,605 (**a**) males and (**b**) females, results are energy adjusted. Tiers are classified according to lower and upper thresholds for nutrients of public health concern: sodium, saturated fats, total fats, and sugars. Lower thresholds: total fat ≤ 3 g/RA, sugars ≤ 6 g/RA, and sodium ≤ 140 mg/RA [5]. Upper thresholds: total fat >10 g/RA, sugars >19 g/RA, sodium >360 mg/RA, and saturated fats >2 g/RA [5]. Tier 1 foods do not exceed lower thresholds for total fat, sugar, and sodium. Tier 2 may exceed one or two lower thresholds but no upper thresholds. For Tier 3, Vegetable and Fruit and Grain Products exceeded all lower threshold and no upper thresholds, or only one upper threshold. For Tier 3 Milk and Alternatives and Meat and Alternatives exceeded all lower threshold and no upper thresholds, or only one upper threshold for total fat, sugars, or sodium. For Tier 4, Vegetable and Fruit and Grain Products exceeded two upper thresholds. For Tier 4, Milk and Alternatives and Meat and Alternatives exceeded upper thresholds for total fat, sugars, or sodium [5].

Figure 1a,b show daily servings from EWCFG subgroups, categorized according to Health Canada’s Tier system for males and females. Among all Vegetable and Fruit subgroups, approximately 90% of intakes were classified as Tier 1 e.g., fruits other than juice, dark green, yellow, and orange vegetables, for all DRI groups (with the exception of fruit juices and vegetable juices). Among all seven Vegetable and Fruit subgroups, potatoes represented the majority of Tier 4 foods in this category (e.g., high in fat, deep fried, or battered) with 56% to 95.5% of servings in males, and 72.2% to 77% of servings in females, respectively for all adults ≥19 years. Within all Meats and Alternatives categorized according to HCST, “poultry” (males: 33.6% to 16.2%, females: 26.8% to 18.97%) and “Beef, game, and organ meats” (males: 21% to 20%, females: 22% to 21.3%) contributed the greatest number of Tier 1–3 servings in adult diets. The majority of foods consumed from eggs and poultry were Tier 2. Furthermore, the top two subgroups with the largest number of Tier 3 foods were “beef, and organ meats” followed by “other meats,” examples of which include deli meats, meats high in fat and sodium etc. Across all age groups, nearly all foods from processed meats were categorized as Tier 4 (males: 53% to 47.2%, females: 52.4% to 42%). Across all ages, the majority of servings within the subgroup “milk and fortified beverages”, which are those low in fat and sugars, were categorized as Tier 2 (males: 40.6% to 54.7%, females: 41.1% to 44.8%). Foods identified as “other milks and alternatives” (e.g., whole milks, flavored drinks, milk-based desserts and most cheeses) were mainly Tier 3 food categories (males: 82.9% to 76.2%, females: 82.6% to 76.5%; Figure 1a,b).

For Canadian adults, 75% to 80% of daily calories came from foods categorized in Tiers 1–3 (Table 2). In total, 23% to 26%, and 21% to 23% of kcal/day were from Tier 4 foods and “other foods”, (i.e., high and low-calorie beverages, alcohol, high fat and/or sugar foods, saturated fats) for adult men and women, respectively. Substantial portions of daily calories were derived from high fat and/or sugar foods; examples of these foods include cheesecakes, pastries, candies etc. In general, men consumed more calories from this food category, with a decrease in consumption with older age groups. The second largest contributor to daily calories in the “other” category were alcoholic beverages, more than high calorie beverages; daily intake of alcohol for males were more than double than for females across all ages (with the exception of 51–70 years).

Individuals in the CCHS were categorized into quartiles (Table 3). Quartile (Q) 1 represents “compliers”, individuals with daily intake of <95% energy from Tier 4 and “other foods”. Q2 and Q3 with 9.95% to 21.05% and 21.06% to 34.65% of daily energy intake from Tier 4 and “others foods” respectively, represent “intermediate compliers”. “Non-compliers”, i.e., those who consumed the highest amount of Tier 4 and “other foods”, as indicated by Q4, represented individuals with >34.65% daily energy from Tier 4 and “other foods”. Mean energy intake adjusted for age, sex, and misreporting ranged from 2183 to 2362 kcal/day, across Q1–Q4 indicating an increase in daily energy across quartiles; *p*-value <0.001. Analysis of intakes show (Q1–Q4) percent of daily energy from nutrients of public health concern increased for fat (31.4% to 33.7%) and saturated fats (9.3% to 11.5%), and decreased for fiber (12.1 to 7.1 g/1000 kcal), *p*-value <0.001; reported intakes of sodium remained high across all quartiles. Intake of vitamin A and D (RE/1000 kcal), vitamin C and calcium, potassium, iron, and zinc (mg/1000 kcal) decreased across Q1–Q4 (Table 3). Body Mass Index (BMI, kg/m^2^) increased from 27.5 to 28.4 kg/m^2^ across Q1–Q4 (compliers–non-compliers; Table 4). As energy intake from Tier 4 and “other foods” increased (Q1–Q4), mean age across quartiles decreased slightly, implying older adults tended to consume less daily energy from unhealthy foods. Smoking was also shown to increase among respondents in Q1–Q4. Furthermore, results from this survey showed more under-reporting (32% to 48%) than over-reporting (4% to 9%) in energy intake across all quartiles.

## 4. Discussion

This study is the first to our knowledge to examine the nutritional quality of foods consumed by Canadians by applying Health Canada’s Surveillance Tool Tier nutrient profiling system to the CCHS-Nutrition 2015, to provide updated evidence on the nutrient profile of foods consumed by Canadians. After categorizing respondents of the CCHS-Nutrition 2015 into quartiles based on graded levels of consumption of Tier 4 and “other foods” (i.e., foods high in sodium, saturated fats, total fats, and sugar) compliers (defined as the lowest consumers of Tier 4 and “other foods”) had better quality diets in terms of calories, total fat, and saturated fats consumed compared to non-compliers. These findings indicate that non-compliers or individuals consuming diets low in nutrients need to consume more nutrient dense foods and make more “healthful” food choices. However, no meaningful difference in sodium intakes was observed among all quartiles. This suggests that sodium intake in Canadian adults was high in the entire population, supporting the findings published by Health Canada [20]. Additionally, dietary intakes of potassium, vitamin D, calcium, and iron were measurably lower as diet quality became nutritionally poorer. These nutrients were identified as a public health concern due to inadequate nutrient intakes and will be declared on the updated Canadian Nutrition Facts table, under the 2016 regulatory changes [21].

According to our findings, daily calorie intake for adults from Tiers 1–3 has increased since the last cycle of CCHS and consumption of foods categorized as Tier 4 have decreased [6]. Calorie contributions from Tier 4 and “other foods” (recommended to limit, and poor in nutritional quality), showed a slight decline across all age groups of adults <70 years in CCHS 2015 compared to results from CCHS 2004. These findings suggest that Canadians are making better food choices in the types of Vegetable and Fruits and Grain Products. For example, across all DRI groups, the majority of Vegetable and Fruits were categorized as Tier 1 or Tier 2 with the exception of potatoes and fruit juice. Similarly, the majority of Grain Products consumed were classified as Tier 2. However, a significant portion of daily calories are still coming from foods that should be chosen less often or to limit. For example, although some Nuts and Seeds from the Meat and Alternatives food group were categorized as Tier 2, the majority of food choices from this group fell under Tier 3, “foods to choose less often”. Although Nuts and Seeds were recommended by EWCFG, the type of products chosen most often by Canadian adults (e.g., “salted nuts and seeds” or “coated nuts”) are items categorized to “choose less often”. In both cycles of the national nutrition surveys and across almost all DRI age groups, the major contributor to the “other foods” category was “high fat and/or sugary foods” [6]. Although Canadians are consuming fewer calories from this category, the quality of food choices consumed remains poor and relatively consistent between both cycles of the CCHS. Our findings suggest that minimal changes have occurred in the nutritional quality of foods consumed by Canadians in the intervening years between 2004 and 2015, which is concerning in terms of progress in nutrition policies and interventions targeted at improving the Canadian food supply and dietary intakes.

Dietary behaviors have been identified as a modifiable risk factor for the prevention of NCD’s. Not only within Canada, but globally, dietary intakes need to be improved and country specific evaluations are lacking. Initiatives set by the World Health Organization (WHO) to reduce dietary intakes of sodium, sugar, and fats are underway in several countries such as in New Zealand, France, the United Kingdom, and Chile [22,23,24,25,26]. These countries have developed nutrient profiling models to encourage healthful food choices and restrict marketing of unhealthy foods to children. In 2017, France implemented the Nutri-Score as a nutrient profiling model to assess foods based on beneficial (i.e., fruits and vegetables, nuts, and fiber) and nutrients (i.e., dietary fat, sugars, sodium, and energy) nutrients [23,24]. The Nutri-Score identifies healthful foods using a color coded scale ranging from green to red as a form of front-of-package (FOP) labelling system to help consumers make nutritious choices. Another example can be seen in the United Kingdom with the Ofcom model. Prior to updated dietary guidelines in the United Kingdom, the Ofcom model was used to identify a “good” or “acceptable” food, similar to other nutrient profiling models based beneficial nutrients and negative nutrients [23,25]. As of March 2018, the Ofcom model is under review to ensure the nutrient scoring system reflects current dietary guidance and changes to recommended energy intakes, free sugars and sodium [25]. Chile and other countries in South America have introduced and approved front of package warning labels identifying foods high in nutrients of public health concern [26].

The present study is the most recent to investigate both the quality and quantity of dietary intakes of Canadian adults using the 2014 HCST, a nutrient profiling model based on thresholds for sodium, saturated fats, total fats, and sugars. With data from the CCHS 2015, our results examined nutrients, servings, and calorie intake in different Tiers of foods. Through application of a nutrient-profiling model developed by researchers at Health Canada, we were able to produce quantitative data to describe the quality of Canadian dietary intakes relative to nutrients of public health concern. These findings are of importance to Canadians, as they reflect the sum of the changes in the food supply and dietary choices, and can be used to develop strategies and policies to help improve Canada’s food environment (e.g., Canada’s Food Guide to promote healthy eating) and to improve the nutritional quality of the Canadian food supply.

This study has several strengths. CCHS Nutrition 2015 is a nationally representative sample of the Canadian population. Respondents were administered a modified AMPM, which was improved from the CCHS 2004 version. Another improvement was in the food module booklet; in this cycle, figures and diagrams were colored and were identified as “better quality” representing real-life items such as glasses, bowls, plates etc. Another strength of this research is that CCHS 2015 collected socio-demographic and lifestyle characteristics such as measured anthropometric data (e.g., used to calculate BMI), which were incorporated as covariates into our statistical models. Furthermore, we were able to adjust our results for misreporting, thus correcting for under-reporting of socially undesirable foods which has been shown to improve the validity of our findings [14,15,16]. However, caution needs to be exercised when interpreting these results as causal inference is not possible with cross-sectional surveys. 

This study is not without limitations. It is important to acknowledge that changes in dietary intakes between CCHS 2004 and CCHS 2015 may be due to differences in the versions of the CNF (CNF 2015 compared to CNF 2001b) used to assess nutrient composition, rather than improved food choices. Since the last national nutrition survey, reformulation of food products in the Canadian food supply has been encouraged by Health Canada. As a result, foods in this survey may have been re-categorized into different Tiers reflecting improved food choices and accounting for changes in energy intake between both cycles of CCHS. An example reported by Garriguet et al. (2018), indicated that energy contributions from Italian salad dressing and baking chocolate have decreased since the CNF 2001b [16,20].

## 5. Conclusions

Overall Canadian dietary intakes are mainly from Tier 2 and Tier 3 foods. Tier 4 and “other foods” represent between 21% and 26% of daily energy intake for adults, with the majority of consumption coming from high fat and sugar foods and alcohol. Several countries have undergone revisions of their dietary guidelines, such as the USDA 2015–2020 Dietary Guidelines for Americans, The Dietary Guidelines for the Brazilian Population, the Australian Dietary Guidelines (2013), and the United Kingdom [25,27,28,29]. Updated evidence based on consumption of nutrients to limit and on adherence to the recently released 2019 Canada’s Food Guide are warranted to assess the progress towards achieving the WHO “best buy” policies [22]. Results from this study provide such evidence for Canadians, and can be used to improve the nutritional quality of the food supply and develop targeted policies supporting healthier food choices for the population [6,7,15,16,30,31,32,33,34].

## Figures and Tables

**Table 1:** Number of servings per food category presented according to the 2014 Health Canada’s Surveillance Tool (HCST) Tier System nutrient profiling model among Canadians ≥19 years, *N* = 13,605.

Food Groups	Tier	19–30 Years	19–30 Years	31–50 Years	31–50 Years	51–70 Years	51–70 Years	>70 Years	>70 Years
		Males	Females	Males	Females	Males	Females	Males	Females
**(Servings/day)**					Mean (SEM)				
**Vegetables and Fruits**	**Tier 1**	2.2 (0.2)	3 (0.2)	3 (0.2)	3.5 (0.2)	3.4 (0.2)	3.8 (0.1)	3.5 (0.2)	3.4 (0.2)
	**Tier 2**	0.9 (0.2)	0.8 (0.1)	0.7 (0.1)	0.7 (0.1)	0.6 (0.1)	0.6 (0.1)	0.7 (0.1)	0.8 (0.1)
	**Tier 3**	0.4 (0.1)	0.5 (0.1)	0.5 (0.1)	0.5 (0.1)	0.5 (0.1)	0.4 (0.1)	0.5 (0.1)	0.4 (0)
	**Tier 4**	0 (0)	0.1 (0)	0 (0)	0 (0)	0 (0)	0.1 (0)	0 (0)	0.1 (0)
	**Tiers 1–3**	3.6 (0.3)	4.4 (0.2)	4.2 (0.2)	4.8 (0.2)	4.4 (0.2)	4.9 (0.2)	4. 7 (0.2)	4.6 (0.2)
**Total**	**Tiers 1–4**	3.6 (0.3)	4.4 (0.2)	4.2 (0.2)	4.8 (0.2)	4.5 (0.2)	4.9 (0.2)	4.7 (0.2)	4.7 (0.2)
**EWCFG Rec.**		8–10	7–8	8–10	7–8	7	7	7	7
**Grain Products**	**Tier 1**	1.3 (0.2)	1.1 (0.1)	1.1 (0.1)	1.2 (0.1)	0.8 (0.1)	0.9 (0.1)	0.9 (0.1)	0.8 (0.1)
	**Tier 2**	3 (0.2)	3 (0.2)	3.4 (0.1)	2.9 (0.1)	3.4 (0.1)	2.9 (0.1)	3.4 (0.1)	3.2 (0.1)
	**Tier 3**	0.6 (0.1)	0.7 (0.1)	0.6 (0.1)	0.7 (0.1)	0.7 (0.1)	0.8 (0.1)	0.8 (0.1)	0.8 (0.1)
	**Tier 4**	0.3 (0.1)	0.5 (0.1)	0.5 (0.1)	0.5 (0.1)	0.4 (0.1)	0.5 (0.1)	0.5 (0.1)	0.6 (0.1)
	**Tiers 1–3**	5.0 (0.3)	4.8 (0.2)	5.1 (0.2)	4.8 (0.2)	4.9 (0.2)	4.6 (0.1)	5.1 (0.2)	4.9 (0.1)
**Total**	**Tiers 1–4**	5.4 (0.3)	5.3 (0.2)	5.6 (0.2)	5.3 (0.2)	5.3 (0.2)	5.1 (0.2)	5.6 (0.2)	5.5 (0.2)
**EWCFG Rec.**		8	6–7	8	6–7	7	6	7	6
**Milk and Alternatives**	**Tier 1**	0.1 (0)	0.1 (0)	0.1 (0)	0.2 (0)	0.2 (0)	0.2 (0)	0.2 (0)	0.2 (0)
	**Tier 2**	0.3 (0.1)	0.4 (0.1)	0.3 (0)	0.4 (0)	0.3 (0)	0.4 (0)	0.5 (0)	0.4 (0)
	**Tier 3**	0.9 (0.1)	1 (0.1)	0.7 (0.1)	0.8 (0.1)	0.7 (0.1)	0.8 (0.1)	0.7 (0.1)	0.9 (0.1)
	**Tier 4**	0.1 (0)	0.1 (0)	0.1 (0)	0.1 (0)	0 (0)	0.1 (0)	0.1 (0)	0.1 (0)
	**Tiers 1–3**	1.3 (0.1)	1.5 (0.1)	1.1 (0.1)	1.4 (0.1)	1.2 (0.1)	1.5 (0.1)	1.4 (0.1)	1.6 (0.1)
**Total**	**Tiers 1–4**	1.3 (0.1)	1.6 (0.1)	1.2 (0.1)	1.5 (0.1)	1.2 (0.1)	1.5 (0.1)	1.5 (0.1)	1.7 (0.1)
**EWCFG Rec.**		2	2	2	2	3	3	3	3
**Meat and Alternatives**	**Tier 1**	0.1 (0)	0.1 (0)	0.2 (0)	0.1 (0)	0.1 (0)	0.1 (0)	0.1 (0)	0.1 (0)
	**Tier 2**	0.6 (0.1)	0.6 (0.1)	0.6 (0.1)	0.6 (0.1)	0.6 (0.1)	0.6 (0.1)	0.4 (0.1)	0.5 (0.1)
	**Tier 3**	1.4 (0.2)	1.1 (0.1)	1.6 (0.1)	1.3 (0.1)	1.6 (0.1)	1.2 (0.1)	1.2 (0.1)	1.1 (0.1)
	**Tier 4**	0.6 (0.1)	0.3 (0.1)	0.3 (0.1)	0.3 (0.1)	0.3 (0.1)	0.3 (0.1)	0.4 (0.1)	0.3 (0.1)
	**Tiers 1–3**	2.4 (0.2)	1.8 (0.1)	2.4 (0.1)	2.0 (0.1)	2.3 (0.1)	1.9 (0.1)	1.7 (0.1)	1.7 (0.1)
**Total**	**Tiers 1–4**	2.7 (0.2)	2.1 (0.1)	2.6 (0.1)	2.3 (0.1)	2.6 (0.1)	2.2 (0.1)	2.1 (0.1)	2.0 (0.1)
**EWCFG Rec.**		3	2	3	2	3	2	3	2

Tiers are classified according to lower and upper thresholds for nutrients of public health concern: sodium, saturated fats, total fats, and sugars. Lower thresholds: total fat ≤3 g/RA, sugars ≤6 g/RA, and sodium ≤140 mg/RA [5]. Upper thresholds: total fat >10 g/RA, sugars >19 g/RA, sodium >360 mg/RA, and saturated fats >2 g/RA [5]. Tier 1 foods do not exceed lower thresholds for total fat, sugar and sodium. Tier 2 may exceed one or two lower thresholds but no upper thresholds. For Tier 3, Vegetable and Fruit and Grain Products exceed all lower threshold and no upper thresholds, or only one upper threshold. For Tier 3, Milk and Alternatives and Meat and Alternatives exceed all lower threshold and no upper thresholds, or only one upper threshold for total fat, sugars, or sodium. For Tier 4, Vegetable and Fruit and Grain Products exceed two upper thresholds. For Tier 4, Milk and Alternatives and Meat and Alternatives exceed upper thresholds for total fat, sugars, or sodium [5]. Data is presented as mean (SEM: standard error of the mean). Servings per/day are classified according to EWCFG: Eating Well with Canada’s Food Guide. Rec: Recommendations. Results are weighted, nationally representative and energy adjusted.

**Table 2:** Weighted analysis of energy contribution from Tier 1–3 and Tier 4 (foods recommended to limit) and “other foods” not recommended by Canada’s Food Guide.

	Men, 19–30 Years	Women, 19–30 Years	Men, 31–50 Years	Women, 31–50 Years	Men, 51–70 Years	Women, 51–70 Years	Men, ≥70 Years	Women, ≥70 Years
Variable (kcal/day)	Mean (SEM)	Mean (SEM)	Mean (SEM)	Mean (SEM)	Mean (SEM)	Mean (SEM)	Mean (SEM)	Mean (SEM)
								
Tiers 1+2+3	1598 (54)	1169 (34)	1544 (31)	1171 (23)	1435 (27)	1102 (15)	1215 (19)	1015 (18)
Tiers 4	246 (42)	122 (15)	175 (12)	108 (11)	157 (10)	118 (7)	162 (14)	119 (9)
	**Other Food/Beverages**							
Alcoholic Beverages	119 (21)	54 (9)	121 (12)	46 (4)	125 (10)	72 (8)	89 (8)	32 (4)
Beverages, High calorie (≥40 kcal/100g)	99 (12)	51 (6)	61 (6)	29 (2)	38 (3)	23 (3)	27 (3)	20 (3)
Beverages, Low calorie (<40 kcal/100 g)	37 (10)	22 (4)	24 (2)	19 (2)	21 (2)	16 (2)	16 (2)	16 (1)
High fat and/ or sugar foods	133 (15)	113 (8)	145 (11)	105 (8)	128 (7)	113 (8)	117 (7)	86 (5)
Meal replacements	4 (3)	3 (1)	1(1)	1 (1)	2 (1)	4 (2)	5 (1)	3 (1)
Saturated and/or trans fats and oils	47 (7)	36 (6)	43 (4)	42 (4)	47 (4)	36 (3)	54 (5)	37 (2)
Uncategorized (ingredients, seasonings, and unprepared foods)	30 (7)	12 (2)	19 (3)	16 (2)	15 (3)	11 (1)	9 (1)	7 (1)
Unsaturated fats and oils	84 (10)	55 (4)	74 (4)	59 (3)	89 (5)	62 (3)	70 (4)	57 (3)
Supplements	6 (3)	2 (1)	2 (1)	4 (1)	1 (0.4)	5 (3)	0.04 (0.04)	0.47 (0.3)
Total energy from Tier 4 and “other foods” and beverages (kcal/day)	681 (62)	397 (21)	568 (21)	350 (14)	516 (20)	378 (15)	464 (19)	310 (13)
Total energy from Tier 4 and “other foods” and beverages (%)	26 (2)	23 (1)	24 (1)	21 (1)	23(1)	22(1)	24 (1)	21 (1)

Tiers are classified according to lower and upper thresholds for nutrients of public health concern: sodium, saturated fats, total fats, and sugars. Lower thresholds: total fat ≤3 g/RA, sugars ≤6 g/RA, and sodium ≤140 mg/RA [5]. Upper thresholds: total fat >10 g/RA, sugars >19 g/RA, sodium >360 mg/RA, and saturated fats >2 g/RA [5]. Tier 1 foods do not exceed lower thresholds for total fat, sugar and sodium. Tier 2 may exceed one or two lower thresholds but no upper thresholds. For Tier 3, Vegetable and Fruit and Grain Products exceed all lower threshold and no upper thresholds, or only one upper threshold. For Tier 3, Milk and Alternatives and Meat and Alternatives exceed all lower threshold and no upper thresholds, or only one upper threshold for total fat, sugars, or sodium. For Tier 4, Vegetable and Fruit and Grain Products exceed two upper thresholds. For Tier 4, Milk and Alternatives and Meat and Alternatives exceed upper thresholds for total fat, sugars, or sodium. Results are presented as mean (SEM: standard error of the mean).

**Table 3:** Weighted analysis of nutrient intakes by compliers, intermediates, and non-compliers based on the percentage of energy consumed from Tier 4 and “other food” and beverages among Canadian (>19 years, *N* = 13,605), adjusted for age, sex, and misreporting status (under-reporter, plausible reporter, and over-reporters).

	Compliers (Q1)	Intermediates (Q2)	Intermediates (Q3)	Non-Compliers (Q4)	*p*-Value
	<9.95% Energy	9.95–21.05% Energy	21.06–34.62% Energy	>34.62% Energy	
Nutrients	Mean (SEM)	Mean (SEM)	Mean (SEM)	Mean (SEM)	
Energy (kcal/day)	2183 (20.2)	2269 (19.7)	2287 (19.8)	2362 (22.8)	<0.0001
Fat (% Energy)	31.4 (0.4)	33.3 (0.34)	33.2 (0.30)	33.7 (0.37)	<0.0001
Saturated fat (% Energy)	9.3 (0.15)	10.7 (0.15)	11.2 (0.15)	11.5 (0.18)	<0.0001
Monounsaturated fat (% Energy)	12.15 (0.23)	12.44 (0.14)	12.41 (0.14)	12.27 (0.16)	0.5836
Polyunsaturated fat (% Energy)	7.0 (0.14)	7.2 (0.17)	6.8 (0.11)	7.0 (0.16)	0.1262
Carbohydrates (% Energy)	48.8 (0.44)	46.8 (0.4)	47.1 (0.35)	44.7 (0.43)	<0.0001
Protein (% Energy)	19.1 (0.25)	17.7 (0.21)	15.7 (0.17)	14.3 (0.16)	<0.0001
Alcohol (% Energy)	0.68 (0.16)	2.23 (0.2)	3.96 (0.24)	7.40 (0.4)	<0.0001
Dietary fiber (g/1000 kcal)	12.1 (0.2)	9.7 (0.17)	9.0 (0.13)	7.1 (0.19)	<0.0001
Vitamin A (RE/1000 kcal)	409 (13)	384 (16)	337 (10)	293 (13)	<0.0001
Vitamin D (RE/1000 kcal)	3.03 (0.10)	2.86 (0.12)	2.36 (0.07)	2.21 (0.09)	<0.0001
Thiamin (mg/1000 kcal)	0.95 (0.02)	0.88 (0.01)	0.84 (0.01)	0.71 (0.01)	<0.0001
Niacin (mg/1000 kcal)	23.46 (0.32)	22.37 (0.36)	19.70 (0.21)	18.46 (0.25)	<0.0001
Riboflavin (mg/1000 kcal)	1.11 (0.02)	1.08 (0.01)	1.01 (0.01)	0.94 (0.01)	<0.0001
Vitamin B6 (ug/1000 kcal)	1.07 (0.02)	0.94 (0.02)	0.84 (0.01)	0.79 (0.02)	<0.0001
Folate (ug/1000 kcal)	141 (3.5)	121 (2)	110 (1.8)	97 (4)	<0.0001
Vitamin B12 (ug/1000 kcal)	2.35 (0.08)	2.44 (0.10)	2.05 (0.07)	1.95 (0.11)	0.0003
Vitamin C (mg/1000 kcal)	65.30 (2.34)	56.13 (2.04)	50.33 (1.90)	43.32 (3.23)	<0.0001
Calcium (mg/1000 kcal)	471.23 (8.39)	452.09 (8.61)	410.44 (6.92)	341.25 (6.09)	<0.0001
Phosphorus (mg/1000 kcal)	780.75 (9.08)	720.57 (8.33)	651.49 (6.77)	591.97 (7.48)	<0.0001
Potassium (mg/ 1000 kcal)	1700.91 (18.99)	1534.07 (17.25)	1424.02 (15.63)	1263.46 (19.97)	<0.0001
Sodium (mg/ 1000 kcal)	1479.31 (25.81)	1470.08 (21.14)	1482.01 (19.44)	1382.06 (17.49)	0.0002
Magnesium (mg/ 1000 kcal)	195.31 (2.56)	174.81 (3.23)	158.99 (1.19)	140.76 (2.17)	<0.0001
Iron (mg/ 1000 kcal)	7.49 (0.11)	6.85 (0.09)	6.55 (0.07)	5.66 (0.08)	<0.0001
Zinc (mg/ 1000 kcal)	6.45 (0.10)	5.92 (0.08)	5.33 (0.08)	4.62 (0.08)	<0.0001

Results are presented as mean (SEM: standard error of the mean). Quartiles are based on the percentage of energy reported for all Tier 4 foods and “other foods” and beverages classified according to lower and upper thresholds for nutrients of public health concern: sodium, saturated fats, total fats, and sugars. Lower thresholds: total fat ≤3 g/RA, sugars ≤6 g/RA, and sodium ≤140 mg/RA [5]. Upper thresholds: total fat >10 g/RA, sugars >19 g/RA, sodium >360 mg/RA, and saturated fats >2 g/RA [5]. Tier 1 foods do not exceed lower thresholds for total fat, sugar and sodium. Tier 2 may exceed one or two lower thresholds but no upper thresholds. For Tier 3, Vegetable and Fruit and Grain Products exceed all lower threshold and no upper thresholds, or only one upper threshold. For Tier 3, Milk and Alternatives and Meat and Alternatives exceed all lower threshold and no upper thresholds, or only one upper threshold for total fat, sugars, or sodium. For Tier 4, Vegetable and Fruit and Grain Products exceed two upper thresholds. For Tier 4, Milk and Alternatives and Meat and Alternatives exceed upper thresholds for total fat, sugars, or sodium. Tier 4 and “other foods” have no Canada’s food guide serving size recommendation. Compliers (Q1) are identified as the lowest 25% of the population with reported intakes from Tier 4 and “other foods” and beverages. Individuals categorized as “Intermediates” (Q2 and Q3) are those with intakes in the interquartile range. Non-compliers (Q4) represent 25% of the population with the highest intakes from Tier 4 and “other foods”.

**Table 4:** Weighted analysis of characteristics of compliers, intermediates, and non-compliers based on the percentage of energy from Tier 4 foods and “other foods” and beverages among Canadian adults (≥19 years, *N* = 13,605).

	Compliers (Q1)	Intermediates (Q2)	Intermediates (Q3)	Non-Compliers (Q4)	*p*- Value
	<9.95% Energy	9.95–21.05% Energy	21.05–34.65% Energy	>34.65% Energy	
Characteristics	Mean (SEM)	Mean (SEM)	Mean (SEM)	Mean (SEM)	
					
Age (years)	50.7 (0.50)	48.2 (0.59)	49.6 (0.51)	49.2 (0.56)	0.0326
Sex (%)					
Males	47.7 (1.50)	44.4 (1.60)	53.5 (1.60)	55.3 (1.70)	
Females	52.4 (1.50)	55.6 (1.60)	46.5 (1.60)	44.7 (1.70)	0.0001
BMI (kg/m^2^)	27.5 (0.19)	27.6 (0.18)	28.1 (0.19)	28.4 (0.21)	0.0031
Physical Activity					
Sedentary	64.2 (1.72)	63.9 (1.76)	68 (1.69)	65.9 (1.75)	0.2354
Active	18 (1.22)	18.2 (1.22)	15.6 (1.12)	16.9 (1.19)	
Misreporting					
Under-reporter (%)	49.8 (1.76)	34.2 (1.72)	32 (1.41)	28.8 (1.53)	<0.0001
Over-reporters (%)	4 (0.37)	7 (0.76)	8 (0.64)	9 (0.76)	
Smoking Status					
Daily Smoker (%)	8.7 (0.91)	13.4 (1.36)	13.5 (1.20)	18 (1.35)	<0.0001
Never Smoked (%)	88 (1.19)	81.8 (1.73)	81.7 (1.42)	76.1 (1.53)	

Results are presented as mean (SEM: standard error of the mean) adjusted for age and sex. Quartiles are based on the percentage of energy reported for all Tier 4 foods and “other foods” and beverages classified according to lower and upper thresholds for nutrients of public health concern: sodium, saturated fats, total fats, and sugars. Lower thresholds: total fat ≤3 g/RA, sugars ≤6 g/RA, and sodium ≤140 mg/RA [5]. Upper thresholds: total fat >10 g/RA, sugars >19 g/RA, sodium >360 mg/RA, and saturated fats >2 g/RA [5]. Tier 1 foods do not exceed lower thresholds for total fat, sugar and sodium. Tier 2 may exceed one or two lower thresholds but no upper thresholds. For Tier 3, Vegetable and Fruit and Grain Products exceed all lower threshold and no upper thresholds, or only one upper threshold. For Tier 3, Milk and Alternatives and Meat and Alternatives exceed all lower threshold and no upper thresholds, or only one upper threshold for total fat, sugars, or sodium. For Tier 4, Vegetable and Fruit and Grain Products exceed two upper thresholds. For Tier 4, Milk and Alternatives and Meat and Alternatives exceed upper thresholds for total fat, sugars, or sodium. Tier 4 and “other foods” have no Canada’s food guide serving size recommendation. Compliers (Q1) are identified as the lowest 25% of the population with reported intakes from Tier 4 and “other foods” and beverages. Individuals categorized as “Intermediates” (Q2 and Q3) are those with intakes in the interquartile range. Non-compliers (Q4) represent 25% of the population with the highest intakes from Tier 4 and “other foods”. BMI represents: body mass index.
